# Risk of short-term cardiovascular disease in relation to the mode of delivery in singleton pregnancies: a retrospective cohort study

**DOI:** 10.1016/j.eclinm.2024.102851

**Published:** 2024-09-26

**Authors:** Gabriella Lobitz, Emily B. Rosenfeld, Rachel Lee, Deepika Sagaram, Cande V. Ananth

**Affiliations:** a Department of Obstetrics, Gynecology, and Reproductive Sciences, Rutgers Robert Wood Johnson Medical School, New Brunswick, NJ, USA; b Division of Maternal-Fetal Medicine, Department of Obstetrics, Gynecology, and Reproductive Sciences, Rutgers Robert Wood Johnson Medical School, New Brunswick, NJ, USA; c Division of Epidemiology and Biostatistics, Department of Obstetrics, Gynecology, and Reproductive Sciences, Rutgers Robert Wood Johnson Medical School, New Brunswick, NJ, USA; d Cardiovascular Institute of New Jersey, Rutgers Robert Wood Johnson Medical School, New Brunswick, NJ, USA; e Department of Biostatistics and Epidemiology, Rutgers School of Public Health, Piscataway, NJ, USA; f Department of Medicine, Rutgers Robert Wood Johnson Medical School, New Brunswick, NJ, USA

**Keywords:** Cardiovascular disease, Caesarean delivery, Vaginal delivery, Stroke

## Abstract

**Background:**

Cardiovascular disease (CVD) is increasing in prevalence and affects up to 4% of pregnancies in otherwise healthy persons. The specific factors that drive the development of CVD in pregnant people are poorly characterised. This study aimed to determine whether the mode of delivery in singletons affects the risk of cardiovascular morbidity and mortality within one year in patients without prior CVD.

**Methods:**

We designed a retrospective cohort study utilising the Nationwide Readmissions Database (NRD) to identify singleton delivery hospitalisations in the United States from Jan 1, 2010 to Nov 30, 2018. *International Classification of Disease* (ICD) versions 9 and 10 codes were used to identify patients with readmission for CVD within the calendar year of index delivery. Patients aged 15–54 who underwent a singleton vaginal or caesarean delivery were included. Patients with pre-existing CVD hospitalisations before or during delivery, ectopic pregnancies, or abortive outcomes were excluded. Participant data was retrieved from the NRD database. The primary outcome was hospital readmission, defined by ICD 9 and 10 codes for fatal or non-fatal CVD in the same calendar year as delivery. Cox proportional hazard regression models were used to adjust for confounders. These included maternal age, hospital bed size, hospital type, hospital teaching status, income quartile, insurance, and year of delivery. Additional sub-analyses were performed adjusting for hypertensive disorders of pregnancy and diabetes mellitus.

**Findings:**

Of the 14,179,299 singleton deliveries, 32% (*n* = 4,553,492) underwent a caesarean. CVD readmissions occurred in 255.2 per 100,000 (*n* = 11,710) caesarean deliveries compared with 133.9 per 100,000 (*n* = 12,507) vaginal deliveries (rate difference [RD], 121.4, 95% confidence interval [CI], 114.8–127.9; hazard ratio [HR] adjusted for all confounders including hypertensive disorders of pregnancy and diabetes mellitus was 1.42, 95% CI 1.35–1.50). This association was highest in the first 0–29 days following delivery (HR 1.68, 95% CI 1.59–1.78). The risk of readmission for CVD persisted for one year.

**Interpretation:**

These findings suggest that caesarean delivery of singletons is associated with a higher risk of cardiovascular morbidity in patients without pre-existing CVD. This risk was highest in the first month but remained elevated for one year after delivery. These findings add to the accumulating evidence that undergoing caesarean delivery may have long-standing health implications and support the extension of the post-partum surveillance period. Limitations of this study include the lack of adjustment for body mass index, race, and parity. We were also unable to determine the reason for the caesarean delivery.

**Funding:**

None.

**Research in context**

**Evidence before this Study**

We searched PubMed and Google Scholar for all studies published on or before June 1, 2023, using the search terms mode of delivery, risk, cardiovascular, vaginal, caesarean or stroke. We also searched for references in relevant papers. Previous observational studies indicated an increased risk of severe maternal morbidity and mortality, including cardiovascular disease (CVD) complications in patients that underwent caesarean deliveries. Overall, data regarding CVD risk and mode of delivery is limited, with existing studies having a limited follow-up period and a small cohort size.

**Added value of this study**

This large study underscores that caesarean delivery of singletons may be associated with a higher risk of cardiovascular disease morbidity in patients without pre-existing CVD. Additionally, this risk was persistent for at least one year following the index delivery.

**Implications of all the available evidence**

Patients who undergo caesarean delivery may be at increased risk for severe complications, particularly CVD morbidity, for at least one year after delivery. These findings should be interpreted cautiously, as we could not adjust for BMI, race, and parity, and the reason for caesarean delivery could not be ascertained. Our findings support an extended and more thorough postpartum observation period, particularly for patients at higher risk.

## Introduction

Cardiovascular disease (CVD) complications occur in up to 4% of all pregnancies in patients without prior disease. They are responsible for over a quarter (27%) of all maternal deaths in the United States.^1^ Patients who experience cardiac complications in pregnancy are at an increased risk of long-term CVD complications. A large cohort study performed in Canada showed that patients with a cardiac complication during pregnancy have a higher long-term risk of CVD hospitalisation in the future than those who experienced other complications of pregnancy, such as a stroke or acute renal failure.^2^ Several factors place pregnant patients at an increased risk of experiencing CVD morbidity and mortality, including Black race, increasing age (specifically over age 40), obesity, tobacco use, and alcohol use.^3^, ^4^, ^5^, ^6^

In reproductive-aged females, the mode of delivery has also been shown to affect their CVD risk. Evidence shows that caesarean delivery confers an increased risk of overall maternal morbidity and mortality.^7^ One study showed that caesarean delivery tripled the risk of severe maternal morbidity and doubled the risk of maternal mortality when compared to vaginal delivery.^8^ The biological basis of this association is not well understood. Still, it may be due to several factors associated with surgery, including the release of cytokines, administration of anaesthesia, and significant fluid shifts.^9^, ^10^, ^11^ While there is some evidence that caesarean delivery increases the risks of maternal morbidity and mortality, there is a dearth of data regarding the relation of caesarean delivery to CVD morbidity and mortality. A population-based study in Sweden reported that the risk for CVD complications doubled among those who underwent caesarean delivery.^12^ This study was limited to complications that occurred within 42 days of delivery and only captured 1267 CVD-related events.

In the United States in 2022, almost a third of patients were delivered via caesarean and thus, it is important to understand the impact this mode of delivery may have on a patient's health.^13^ To address this knowledge gap, we designed a large, retrospective cohort study in the US to examine if the mode of delivery in singletons affects a patient's risk of experiencing short-term (within the calendar year of delivery) CVD morbidity or mortality. We hypothesise that caesarean delivery will confer an increased CVD morbidity and mortality risk.

## Methods

### Data sources

In this retrospective cohort study, we utilised the Nationwide Readmissions Database (NRD) from 2010 to 2018 and included over 14 million unweighted and 30 million weighted delivery discharges. The data is sourced from and maintained by the Healthcare Cost and Utilization Project of the Agency for Healthcare Research and Quality.^14^^,^^15^ The NRD database includes discharged patients with and without repeat hospital visits and in-hospital deaths within a state up to the last calendar date of discharge year (i.e., December 31st). The NRD database included inpatient data from 18 states in 2010 (1809 hospitals, 13,907,610 unweighted discharges, and 37,284,093 weighted discharges). It expanded in 2018 to include 28 states (2430 hospitals, 17,686,511 unweighted discharges, and 35,460,557 weighted discharges), representing 56% of the United States population. We used sample weights to allow for extrapolation to nationwide representation.^15^ Before release, the NRD removed all personal data identifiers so approval from our institution's ethical review board and informed consent of participants was not required. The Strengthening the Reporting of Observational Studies in Epidemiology^16^ (STROBE) guidelines for cohort studies were followed for this study.

### Cohort composition

**Fig. 1:**
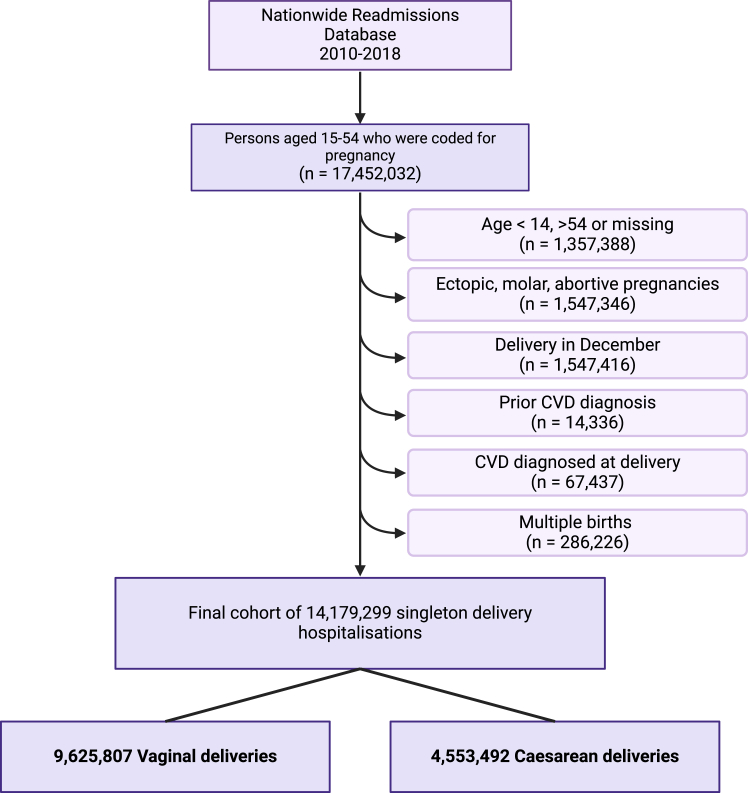
Flow diagram of participant inclusion. CVD, Cardiovascular disease.

We identified females ages 15–54 who underwent a hospitalisation that resulted in a singleton delivery from January 1, 2010 to November 30, 2018. All deliveries were determined using the *International Classification of Disease* [ICD] 9 (2010 to third quarter 2015) and 10 (2015 fourth quarter to 2018) diagnoses and procedure codes. Deliveries in December (all years) were not included as it would not allow 30 days of follow-up time (the minimum period in our study; see below). Verified patient linkage numbers and a timing variable between each hospital admission for each patient provided by the NRD enabled us to distinguish between separate admissions before or after the delivery discharge date. The database also allowed for the identification of transfers of patients between facilities as separate from the new admissions. Patients with ectopic, molar, or abortive outcomes were excluded (Fig. 1).

Conditions considered as CVD included ischaemic heart disease, atherosclerotic heart disease, acute myocardial infarction, hypertensive heart disease, heart failure, cardiomyopathy, cardiac arrhythmias, or stroke. CVD mortality in our study was defined as NRD-identified in-hospital deaths coupled with CVD diagnosis codes at the time of hospitalisation.

Patients with a CVD-coded hospitalisation before and during the delivery admission were excluded. Patients with CVD-related readmissions (defined by the ICD 9 and 10 codes in Supplemental Table S1) that were separate readmissions (determined by the ICD 9 and 10 codes in) from the index delivery admission were identified between January and December of each calendar year.

### Exposure

All patients who underwent caesarean delivery, excluding those with a CVD-coded hospitalisation before or at the time of delivery, were considered to have the exposure. Additionally, information was collected regarding the type of caesarean delivery, whether it was a primary or repeat surgery, and whether the patient was labouring before the caesarean. Labouring caesarean deliveries included any deliveries coded as having had an arrest of labour at any stage as defined by ACOG or acute signs of foetal or maternal distress.^17^

### Outcomes

The primary outcome was any CVD-related readmission within the calendar year following the index delivery. Secondary outcomes were assessed by CVD type, CVD mortality, and heart disease and stroke morbidity and mortality. The causal effect of interest in this study was the risk of CVD complications after delivery and whether it differed based on the mode of delivery.

### Covariates

Potential confounders, including patient characteristics and hospital-related factors, were examined and identified using the ICD 9 and 10 codes (Supplemental Table S1). Patient characteristics included maternal age, insurance payer, median income quartile derived from patient's residence and varied by year, and year of delivery hospitalisation.^18^ Hospital factors included teaching status, bed size, and hospital type. An additional analysis was performed, adjusting for all covariates as in the initial model, as well as hypertensive disorders of pregnancy (HDP) and diabetes mellitus (DM).

### Statistical analysis

Rates of CVD, heart disease, and stroke among those who underwent delivery are expressed as rates per 100,000 deliveries. The rate difference (RD) between readmission for CVD based on the mode of delivery was examined with a 95% confidence interval. Associations between mode of delivery and CVD readmission and mortality were determined using Cox proportional hazard regression models with CVD readmission as the event and person-months (time between hospitalisation for delivery and CVD readmission) as the time scale.^19^ To account for the fact that the NRD only provides the month and year of delivery at readmission, a random integer for the day of the event was generated based on the uniform distribution of the cohort.

To address confounding bias, all models were adjusted for maternal age (grouped by 5-year categories as <20, 20–24, 25–29, 30–34, 35–39, 40–44, 45–54), hospital bed size (classified as small, medium or large), hospital type (government non-federal, private non-profit, private investment owned), teaching status (metro teaching, metro non-teaching, non-metro), income quartile (grouped as low, medium-low, medium–high, high), insurance (categorised as Medicare, Medicaid, private, self-pay, others and unknown) and year of delivery.^20^ A separate analysis was performed following additional adjustments for HDP and DM.

Several sub-analyses compared CVD complication rates between primary and repeat and labouring and non-labouring caesareans. Finally, utilising the Cox proportional hazard model, the risks of CVD hospital readmission, mortality, and stroke were estimated in discrete time intervals during the post-partum period, namely 0–29, 30–59, 60–89, 90–179, and 180–365 days following the index delivery. This analysis was designed to address the potential violation of proportional hazard assumptions. For simplicity, we only report the overall and the 0–29 hazard ratios in the main table, and other time points are provided in the Supplemental Table.

### Sensitivity analysis

We undertook a probabilistic bias analysis given the potential for selection bias of pregnancies without CVD complications before delivery and the likelihood of the effect size estimates being affected by unmeasured confounding. The selection bias probability was fixed at 0.96 (4% of pregnancies affected by CVD).^1^ For unmeasured confounding, we assumed nondifferential prevalence estimates of confounders among caesarean and vaginal deliveries to be between 0.40 and 0.45 under a uniform distribution, the confounder-to-outcome relative risk to be between 1.0 and 10.0 under a uniform distribution based off clinically plausible values, and the correlation between the exposure-specific confounder prevalences to be 0.3. We generated 500,000 simulation patterns and reported the median bias-corrected rate ratio (RR) and 95% CI. It should be noted that this bias analysis was not adjusted for any of the aforementioned confounders.

### Role of the funding source

There was no funding for this study. All authors had full access to the data used in this study and had final responsibility for the decision to submit for publication.

## Results

Of the 14,179,299 eligible deliveries included in the analysis, nearly a third were delivered by caesarean (32.0%; n = 4,553,492; median [IQR], 28.9 [24.4–33.1] years old), and two-thirds were delivered by vaginal delivery (68.0%; n = 9,625,807; median [IQR], 27.2 [22.7–31.4] years old). Metropolitan teaching hospitals were the setting for 58.8% of all deliveries. The distribution of patient demographic characteristics and hospital factors based on the mode of delivery is shown in Table 1.

**Table 1:** Sociodemographic characteristics of patients without prior cardiovascular disease: Nationwide Readmissions Database, 2010–2018.

	Total delivery hospitalisations number (weighted %_col_)	Vaginal delivery number (weighted %_col_)	Caesarean delivery	
			Number (weighted %_col_)	Weighted %_row_
**Number of delivery hospitalisations**	14,179,299 (100.0)	9,625,807 (100.0)	4,553,492 (100.0)	32.0
**Type of caesarean**				
Primary	–	–	2,087,124 (46.0)	14.7
Repeat	–	–	2,466,368 (54.0)	17.3
**Labour status**				
Labouring	–	–	1,850,396 (39.7)	12.7
Non-labouring	–	–	2,701,510 (60.3)	19.3
**Age at delivery (years)**				
<20	871,791 (7.1)	686,854 (8.2)	184,937 (4.7)	21.1
20–24	3,044,303 (22.0)	2,229,317 (23.5)	814,986 (18.5)	27.0
25–29	4,050,015 (28.8)	2,812,681 (29.3)	1,237,334 (27.6)	30.7
30–34	3,848,099 (26.6)	2,524,427 (25.6)	1,323,672 (28.7)	34.5
35–39	1,915,072 (12.7)	1,140,116 (11.1)	774,956 (16.2)	40.6
40–44	422,412 (2.8)	221,192 (2.12)	201,220 (4.1)	47.7
45–54	27,607 (0.2)	11,220 (0.1)	16,387 (0.3)	58.8
**Hospital bed size**				
Small	1,828,840 (13.5)	1,287,425 (14.0)	541,415 (12.6)	29.7
Medium	3,948,945 (27.5)	2,676,562 (27.5)	1,272,383 (27.5)	31.9
Large	8,401,514 (58.9)	5,661,820 (58.5)	2,739,694 (60.0)	32.6
**Hospital type**				
Government, non-federal	1,827,435 (11.8)	1,228,107 (11.7)	599,328 (12.1)	32.8
Private, not profit	10,482,044 (75.7)	1,165,434 (76.2)	1,316,610 (74.6)	31.6
Private, investment owned	1,869,820 (12.5)	1,232,266 (12.1)	637,554 (13.3)	34.0
**Teaching hospital status**				
Metro, non-teaching	4,629,561 (30.8)	3,130,234 (30.7)	1,499,327 (31.1)	32.3
Metro, teaching	8,359,101 (58.8)	5,670,811 (58.8)	2,688,290 (59.0)	32.1
Non-metro hospital	1,190,637 (10.3)	824,763 (10.5)	365,875 (9.9)	30.8
**Insurance**				
Medicare	96,163 (0.7)	57,437 (0.6)	38,729 (0.9)	40.4
Medicaid	6,062,849 (42.6)	4,162,458 (43.1)	1,900,391 (41.4)	31.1
Private	7,344,324 (51.9)	4,928,871 (51.2)	2,415,453 (53.3)	32.9
Self-pay	217,801 (1.5)	157,959 (1.6)	59,842 (1.3)	27.1
Others	433,491 (3.1)	302,531 (3.2)	130,960 (2.9)	30.0
Unknown	24,671 (0.2)	16,551 (0.2)	8120 (0.2)	32.5
**Income**				
Low	3,761,435 (27.8)	2,535,844 (27.7)	1,225,591 (28.0)	32.3
Medium low	3,473,165 (25.1)	2,365,034 (25.1)	1,108,131 (24.9)	31.8
Medium-high	3,572,404 (24.8)	1,445,076 (25.0)	1,127,328 (24.4)	31.5
High	3,234,944 (21.4)	2,186,850 (21.3)	1,048,094 (21.7)	32.4
Unknown	137,351 (0.9)	93,003 (0.9)	44,348 (0.9)	32.4
**Fetal growth restriction**				
No	13,790,228 (97.2)	9,394,461 (97.5)	4,395,767 (96.5)	31.8
Yes	389,071 (2.8)	231,346 (2.5)	157,725 (3.5)	40.2
**Stillbirth**				
No	14,118,618 (99.6)	9,575,549 (99.5)	4,543,069 (99.8)	32.1
Yes	60,681 (0.4)	50,258 (5.2)	10,423 (0.2)	17.0
**Diabetes mellitus**				
Non-diabetics	13,597,850 (96.3)	9,316,613 (97.1)	4,281,237 (94.5)	31.4
Pre-pregnancy diabetes	80,255 (0.6)	28,471 (0.3)	51,784 (1.2)	64.5
Gestational diabetes	449,506 (0.1)	262,082 (2.5)	187,424 (4.3)	44.2
**Hypertension**				
Normotensive	12,604,673 (88.8)	8,771,764 (91.1)	3,832,909 (84.1)	30.2
Any hypertensive disorder	1,572,659 (11.2)	853,093 (8.9)	719,566 (15.9)	45.7
Chronic Hypertension	255,364 (1.8)	129,074 (1.4)	125,660 (2.8)	49.2
Gestational hypertension	573,138 (4.1)	361,402 (3.8)	211,736 (4.7)	36.9
Mild Preeclampsia	317,306 (2.2)	182,659 (1.9)	134,647 (3.0)	42.4
Severe Preeclampsia	241,128 (1.7)	96,490 (1.1)	144,638 (3.2)	60.0
Eclampsia	9331 (0.1)	3618 (0.04)	5713 (0.1)	61.8
Superimposed Preeclampsia	100,389 (0.7)	37,250 (0.4)	63,139 (1.4)	63.0
Unspecified hypertension	77,970 (0.6)	42,920 (0.5)	35,050 (0.8)	44.7
**Placental abruption**				
No	14,031,307 (99.0)	9,563,622 (99.4)	4,467,685 (98.1)	31.7
Yes	147,992 (1.0)	62,185 (0.6)	85,807 (1.9)	58.0
**Preterm delivery**				
No	13,669,147 (96.1)	9,315,233 (96.5)	4,353,914 (95.4)	31.7
Yes	510,152 (3.9)	310,574 (3.5)	199,578 (4.6)	38.4
**Year of hospitalisation**				
2010	1,406,752 (11.3)	939,694 (11.1)	1,118,022 (11.6)	33.0
2011	1,400,444 (11.2)	937,480 (11.0)	462,964 (11.4)	32.8
2012	1,370,250 (11.1)	917,809 (11.0)	452,441 (11.4)	32.9
2013	1,481,870 (11.0)	1,001,507 (11.1)	480,363 (11.2)	32.2
2014	1,557,816 (11.2)	1,057,676 (11.3)	500,140 (11.2)	31.8
2015	1,743,595 (11.2)	1,190,368 (11.3)	553,227 (11.0)	31.5
2016	1,731,082 (11.2)	1,186,296 (11.3)	544,786 (10.9)	31.3
2017	1,764,630 (11.0)	1,210,550 (11.0)	554,080 (10.7)	31.3
2018	1,722,860 (10.8)	2,229,547 (10.9)	538,433 (10.5)	31.2

The median follow-up for caesarean and vaginal delivery patients was 6.3 (IQR 3.7, 9.2) and 6.3 (IQR 3.7, 9.1) months, respectively (Table 2). Mortality rates from CVD in the vaginal and caesarean delivery groups were 2.9 and 3.8 per 100,000 delivery hospitalisations, respectively (rate difference 1, 95% CI 0.1–1.6). Compared to vaginal delivery (133.9 per 100,000 delivery hospitalisations), there were an excess 121.4 (95% CI 114.8–127.9) non-fatal CVD readmissions in the caesarean delivery (255.2 per 100,000 delivery hospitalisations). Caesarean delivery was also associated with excess readmissions for non-fatal heart disease and stroke.

**Table 2:** Rates of cardiovascular disease mortality and readmission amongst vaginal versus caesarean delivery among patients without pre-existing cardiovascular disease: Nationwide Readmissions Database, 2010–2018.

Cardiovascular disease	Vaginal delivery number (Rate per 100,00)	Caesarean delivery number (Rate per 100,00)	Rate difference (95% confidence interval)
**Number of delivery hospitalisations**	9,625,807	4,553,492	–
**Mortality**			
All-cause	489 (5.1)	395 (8.4)	3.4 (2.3–4.4)
Cardiovascular disease	267 (2.9)	176 (3.8)	0.9 (0.1–1.6)
Heart disease	226 (2.5)	146 (3.1)	0.6 (−0.1 to 1.3)
Stroke	64 (0.7)	51 (1.2)	0.4 (−0.0 to 0.9)
**Non-fatal complications**			
Cardiovascular disease (any)	12,507 (133.9)	11,710 (255.2)	121.4 (114.8–127.9)
Heart disease (any)	10,494 (113.1)	10,353 (226.2)	113.1 (107.7–119.2)
Ischaemic heart disease	1276 (12.8)	1058 (22.4)	9.6 (7.7–11.5)
Atherosclerotic heart disease	591 (7.0)	464 (10.5)	3.5 (2.1–5.0)
Acute myocardial infarction	596 (6.1)	450 (9.6)	3.5 (2.3–4.8)
Hypertensive heart disease	710 (6.8)	975 (19.7)	12.9 (11.4–14.4)
Heart failure	4377 (46.9)	5181 (112.5)	65.5 (61.2–69.8)
Cardiomyopathy	1456 (14.9)	1410 (30.2)	15.2 (13.0–17.4)
Cardiac arrythmias	4993 (54.6)	4098 (90.9)	36.2 (32.5–39.9)
Stroke (any)	2376 (24.6)	1591 (34.0)	9.5 (7.0–11.9)
Ischaemic stroke	1516 (15.4)	1044 (22.5)	7.1 (5.1–9.0)
Haemorrhagic stroke	1013 (10.4)	606 (12.9)	2.5 (1.0–4.0)

Caesarean delivery was associated with a 76% higher risk of CVD morbidity (HR 1.76, 95% CI 1.68–1.84) following adjustment for confounders (Table 3). Further adjustment for HDP and DM showed an attenuated but persistent association (HR 1.42, 95% CI 1.35–1.50) (Table 3). This increased risk was observed for all CVD subtypes and was particularly high for heart failure (HR 2.02, 95% CI 1.85–2.21). Again, when adjusting for HDP and DM as confounders, the risk was attenuated (HR 1.50, 95% CI 1.36–1.65). The risk of cardiomyopathy, a severe CVD complication, was higher in the caesarean group (HR 1.76, 95% CI 1.51–2.05). The risk of overall stroke readmission was also higher among the caesarean delivery group (HR 1.40, 95% CI 1.26–1.56).

**Table 3:** Weighted associations between mode of delivery and hospitalisations for cardiovascular disease: Nationwide Readmissions Database, 2010–2018.

Cardiovascular disease	Adjusted hazard ratio (95% confidence interval)			
	Hospitalisations up to a year		Hospitalisations 0–29 days	
	Adjusted^a^	Adjusted^b^	Adjusted^a^	Adjusted^b^
**Mortality**				
All-cause	1.32 (1.11–1.56)	1.14 (0.96–1.36)	1.69 (1.16–2.45)	1.60 (1.08–2.36)
Cardiovascular disease	1.19 (0.97–1.46)	1.02 (0.83–1.27)	1.67 (1.09–2.57)	1.58 (1.01–2.47)
Heart disease	1.16 (0.93–1.45)	1.00 (0.79–1.27)	1.48 (0.89–2.46)	1.43 (0.84–2.42)
Stroke	1.47 (0.97–2.22)	1.25 (0.81–1.91)	2.31 (1.17–4.55)	2.11 (1.03–4.32)
**Non-fatal complications**				
Cardiovascular disease (any)	1.76 (1.68–1.84)	1.42 (1.35–1.50)	1.99 (1.88–2.10)	1.68 (1.59–1.78)
Heart disease (any)	1.81 (1.72–1.91)	1.46 (1.38–1.54)	2.14 (2.01–2.26)	1.80 (1.69–1.91)
Ischaemic heart disease	1.52 (1.33–1.72)	1.17 (1.02–1.34)	1.50 (1.24–1.82)	1.27 (1.06–1.52)
Atherosclerotic heart disease	1.56 (1.26–1.90)	1.30 (1.06–1.61)	1.76 (1.33–2.33)	1.52 (1.13–2.05)
Acute myocardial infarction	1.34 (1.14–1.59)	1.06 (0.89–1.27)	1.12 (0.87–1.44)	0.99 (0.79–1.25)
Hypertensive heart disease	2.00 (1.57–2.55)	1.31 (0.98–1.74)	2.43 (2.03–2.91)	1.69 (1.39–2.05)
Heart failure	2.02 (1.85–2.21)	1.50 (1.36–1.65)	2.44 (2.25–2.65)	1.95 (1.79–2.12)
Cardiomyopathy	1.76 (1.51–2.05)	1.28 (1.08–1.53)	2.24 (1.89–2.65)	1.79 (1.49–2.15)
Cardiac arrythmias	1.64 (1.54–1.75)	1.45 (1.36–1.54)	1.90 (1.73–2.09)	1.75 (1.59–1.92)
Stroke (any)	1.40 (1.26–1.56)	1.17 (1.05–1.31)	1.19 (1.03–1.38)	1.05 (0.91–1.22)
Ischaemic stroke	1.46 (1.29–1.65)	1.19 (1.03–1.37)	1.33 (1.13–1.57)	1.13 (0.95–1.14)
Haemorrhagic stroke	1.20 (1.03–1.39)	1.14 (0.98–1.32)	0.98 (0.78–1.24)	0.95 (0.75–1.20)

a Hazards ratios were adjusted for the confounding effects of maternal age, hospital bed size, hospital type, hospital teaching status income quartile, insurance, and year of delivery through the Cox proportional hazards regression model.

b Hazards ratios were adjusted for all variables as in the previous model, plus hypertensive disorders of pregnancy and diabetes status.

The risk of overall CVD readmission varied over time in the post-partum period and was highest at 0–29 days following the index delivery (HR 1.99, 95% CI 1.88–2.10) (Table 3). After adjustment for HDP and DM, the association was attenuated (HR 1.68, 95% CI 1.59–1.78). Readmission risk was elevated for all CVD subtypes across all time periods. The associations were generally the strongest in the initial 0–29 days postpartum, with the association being highest for hypertensive heart disease (HR 2.43, 95% CI 2.03–2.91) and heart failure (HR 2.44, 95% CI 2.25–2.65). The associations fluctuated for CVD subtypes, decreasing at 30–59 days postpartum, increasing slightly again at 60–89 days, and decreasing with increasing latency (Supplemental Table S2). Stroke risk exhibited a different pattern, being lowest in the first 0–29 days (HR 1.19, 95% CI 1.03–1.38) and increasing with time, with the strongest association being at 180–365 days postpartum (HR 1.94, 95% CI 1.53–2.46). The Kaplan–Meier curves for CVD morbidity and mortality with caesarean delivery over time are depicted in Supplemental Figures S1 and S2, respectively.

Patients who underwent primary caesarean delivery exhibited a 93% increased risk of overall CVD readmission (HR 1.93, 95% CI 1.82–2.05), slightly higher than those who underwent a repeat caesarean (HR 1.56, 95% CI 1.47–1.65) (Supplemental Table S3). Patients who were labouring before caesarean delivery had a slightly lower risk of overall CVD readmission (HR 1.62, 95% CI 1.53–1.71) when compared to patients who were not labouring before surgery (HR 1.82, 95% CI 1.71–1.93) (Supplemental Table S4).

Finally, CVD mortality risk was increased by 19% in the caesarean delivery group (HR 1.19, 95% CI 0.97–1.46). However, after adjusting for protentional confounders, caesarean delivery was not meaningfully associated with CVD mortality (HR 1.02, 95% CI 0.83–1.27) (Table 3). The main results are summarised in the infographic (Supplemental Figure S3).

### Sensitivity analysis

The results of the probabilistic bias analysis following corrections for selection and unmeasured confounding biases on the mode of delivery slightly attenuated the risk of CVD morbidity up to one year (RR 1.47, 95% CI 1.40–1.55) (Supplemental Table S5). The attenuation of risk was also seen when the probabilistic bias analysis was performed for CVD hospitalisations 0–29 days after delivery (RR 1.59, 95% CI 1.51–1.66). Stroke and all-cause mortality risks for caesarean delivery remained elevated after bias correction.

## Discussion

This large, population-based study shows that caesarean delivery was associated with a 76% increased risk of CVD morbidity when compared to vaginal delivery. The risk was attenuated but remained elevated after adjusting for the previously described confounders as well as HDP and DM. These findings are in line with previous data showing an increased risk of general maternal mortality and severe morbidity with caesarean delivery.^7^^,^^8^ Hazard ratios were elevated across all sub-types of heart disease morbidity as well as stroke. These results were consistent when adjusting for the nature of the caesarean.

Both primary and repeat caesarean deliveries were associated with elevated CVD readmission risk in relation to vaginal delivery. Primary caesareans carried an overall slightly higher risk than repeat caesarean deliveries across all CVD sub-types. We hypothesise that this may be due to other maternal or foetal comorbidities necessitating primary caesarean delivery that may contribute to their risk of CVD complications. Non-labouring caesarean deliveries were also compared. CVD readmission risk was somewhat higher but overall comparable to the non-labouring group. Again, this may be contributable to maternal conditions that necessitate scheduled caesarean delivery (i.e., non-labouring). However, these data contradict the findings of other studies, and further investigation into this sub-category is warranted.^21^

The association of CVD morbidity and caesarean delivery fluctuated over time. Our data shows that CVD risk is generally highest 0–29 days following caesarean and declines with increasing latency from the index delivery. Some subtypes, specifically atherosclerotic heart disease, ischaemic heart disease, cardiomyopathy, acute myocardial infarction, and heart failure, exhibit an increase in risk in the 60 to 89-day postpartum period after an initial decline in the 30 to 59- day period. This pattern is not readily explained or previously demonstrated in the current literature and warrants further investigation. The risk of stroke morbidity exhibited a different pattern. The risk of any non-fatal stroke readmissions, including ischaemic or haemorrhagic types, in relation to caesarean delivery, was lowest in the first 30 days after delivery but increased as time from delivery increased, being highest in the 180–365 days postpartum. This observation echoes that of other studies that found most post-partum strokes (46.7%) occur 12–52 weeks after delivery.^22^ The pathophysiologic drivers of this pattern are unclear. It may indicate the role of more insidious long-term vascular remodelling and metabolic imbalances that persist after caesarean delivery — the actual cause of this delayed stroke risk warrants further investigation.

While the data supports a link between caesarean delivery and CVD morbidity, the biological pathway of this connection is imperfectly described and likely multifactorial. The pathways leading to cardiac events after surgery have been studied in several disciplines, including abdominal and vascular surgery. Surgery induces cytokine and pro-inflammatory marker release, possibly contributing to arterial thrombi formation.^11^^,^^23^^,^^24^ Several aspects of surgical intervention, such as anaesthetic administration, anaemia, and hypothermia, trigger the release of catecholamines and cortisol.^9^^,^^10^ These changes cause elevated blood pressure, heart rate, and possible transient myocardial ischaemia, all of which may contribute to the elevated CVD morbidity and mortality risk observed in our study. Other post-operative factors, including medications for pain control, may also contribute to the observed risk. Non-steroidal anti-inflammatory drugs (NSAIDs) are often prescribed at higher dosages in more frequent intervals to post-operative caesarean patients, and their utilisation is increasing with the push to reduce opioid use.^25^ Data regarding the relative safety of common NSAIDs, such as ibuprofen and diclofenac, remains unclear. Some studies suggest that these medications may also be associated with elevated cardiovascular risk, particularly myocardial infarction.^26^^,^^27^ Additional factors, such as prolonged hypomobility and the acute fluid shifts that accompany caesarean delivery more often than vaginal delivery, are also possible contributors to this pathway.

Cardiac complications of pregnancy have long-term implications for future health outcomes. Previous data showed that patients who experience hypertensive disorders of pregnancy have an increased lifetime risk of developing some form of cardiac disease and may do so at an earlier age.^28^^,^^29^ Our study showed that the risk for hypertensive heart disease increased two-fold when a patient underwent a caesarean delivery compared to those who delivered vaginally, possibly affecting the life-long health of these patients. Additionally, our data shows that patients who undergo caesarean delivery have an approximately 76% increased risk of peripartum cardiomyopathy, with this risk remaining elevated 180 days from the index delivery. One large study assessing the long-term risk of pregnancy complications reported that peripartum cardiomyopathy was associated with the greatest risk of experiencing future cardiovascular disease when compared to other cardiac complications.^2^

Strengths of this study include a large sample size of over 14 million deliveries. Using the NRD allowed for a vast and representative data set. Because of the magnitude of data available through the NRD, our study was able to adjust for many confounders. We performed an additional sub-analysis adjusting for HDP and DM. However, the exact role of these conditions as either a proxy or confounder for caesarean is unclear. HDP and DM, particularly poorly controlled forms of these disease, at times, can be the direct cause for delivery by caesarean. In these cases, to adjust for HDP and DM may result in over-adjustment.

This study has limitations that are inherent to large database studies. Persons with prior CVD were excluded based on evidence of previous hospital admission within the calendar year before delivery for cardiac causes. Due to the nature of this database, if the patient was admitted to the hospital with a CVD diagnosis before the delivery year and their CVD status was not carried over to the delivery admission, they would not have been excluded. The probabilistic bias analysis accounts for such potential selection bias and shows the associations between mode of delivery and CVD risks remain similar. This data set can also not discern the person's parity during index delivery. Prior studies have demonstrated that lifetime CVD risk may vary depending on a patient's parity.^30^ This data set's limitations in evaluating the dose effect of caesarean delivery make it important to acknowledge that the CVD risks associated with caesarean are heterogeneous and dependent on parity. The lack of parity data may affect the sub-analysis comparing primary versus repeat caesarean, as patients undergoing their first caesarean may not be primiparous.

Additionally, this dataset is limited in adjusting for all possible confounders. Body Mass Index (BMI) is a risk factor for developing comorbidities such as diabetes and hypertension, and it is an independent risk factor for delivery by caesarean.^31^^,^^32^ The BMI data obtained from the NRD was incomplete, with more than 75% of patients missing BMI data, and as such, we were unable to adjust for this confounder in our analysis. Additionally, race data was not available in the NRD. Future studies that include race, ethnicity, BMI, and other potential confounders, such as smoking, may better characterise the association between caesarean delivery and CVD. Finally, by nature of the database, the exact indication for undergoing caesarean cannot be determined. For these reasons, the results should be interpreted with caution. However, the bias-corrected rate ratio attempts to account for such unmeasured confounding. Most deliveries in the United States are singletons; thus, this limitation does not severely limit the generalisability of the findings.

Identification of CVD morbidity and mortality was done via ICD-9 and ICD-10 codes. The use of a universal and standardised coding system helps ensure that cohort composition, exposure, and outcome data are accurate to a reasonable extent. However, coding consistency is dependent on human error and may be inconsistent across providers and institutions. Other studies have previously validated the sensitivity and specificity of ICD 9 and 10 codes to detect specific CVD complications. We performed a bias analysis using this data to account for possible errors in coding.^33^, ^34^, ^35^ We therefore suggest that these associations be interpreted with some caution. Errors or omissions in proper ICD coding may also contribute to the low rate of preterm birth observed in this cohort (approximately 3.6%) compared to the reported national average of 10–11%.^36^

In conclusion, this study found that caesarean delivery may be associated with an increased risk of cardiovascular and stroke morbidity. This positive association was observed when controlling for several demographic confounders, types, and indications for caesarean. The association was strongest in the first 30 days after delivery for CVD; however, the risk remained elevated up to 365 days. A better understanding of the risks associated with caesarean delivery is an important factor in patient counselling and decision-making. The persistent elevation of this risk beyond the current standard post-partum period of six weeks supports the need to extend the surveillance period, particularly after caesarean delivery. With one-third of all patients undergoing caesarean delivery, future research regarding the implications of CVD complications in pregnancy is warranted. The next steps in this area of investigation include determining if CVD risk with caesarean delivery is further potentiated in those patients with pre-existing CVD.

## Contributors

Conceptualisation: GL and CVA.

Data curation: RL and CVA. RL and CVA have access to and verify the underlying study data.

Formal Analysis: RL and CVA.

Funding acquisition: Not applicable.

Investigation: GL and CVA.

Methodology: GL, ER, RL, DS and CVA.

Project administration: CVA.

Resources: CVA.

Software: RL and CVA.

Supervision: CVA.

Validation: RL and CVA.

Visualisation: GL, RL, and CVA.

Writing—original draft: GL.

Writing—review & editing: GL, ER, RL, DS and CVA.

## Data sharing statement

The data underlying this article were provided by Healthcare Cost and Utilization Project under license and by permission, and so cannot be shared.

## Declaration of interests

All authors declare no competing interests.
