# Supplementary material for: Risk of short-term cardiovascular disease in relation to the mode of delivery in singleton pregnancies: a retrospective cohort study
Source: eClinicalMedicine. 2024 Sep 26;76:102851. doi: 10.1016/j.eclinm.2024.102851 (PMC11466563; doi:10.1016/j.eclinm.2024.102851)

Risk of short-term cardiovascular disease in relation to the mode of delivery in singleton pregnancies: a retrospective cohort study

Gabriella Lobitz, MD, Emily B. Rosenfeld, Rachel Lee, MS,

Deepika Sagaram, M, Cande V. Ananth

Supplemental Table 1
International Classification of Disease (ICD) 9 and 10 codes for mode of delivery and cardiovascular disease

	ICD-9<br>(2010 to 2015 third quarter)	ICD-10<br>(2015 fourth quarter to 2018)
**Inclusion Criteria**		
Delivery	V27, 650,<br>720*, 721*, 7221*, 7229*,<br>7231*, 7239*, 724*, 726*,<br>7251*, 7252*, 7253*, 7254*,<br>7271*, 7279*,<br>728*, 729*,<br>7322*	Z37, O80,<br>10D07Z3*, 0W8NXZZ*,<br>10D07Z4*, 10D07Z5*,<br>10S07ZZ*, 10D07Z3*,<br>10D07Z4*, 10D07Z5*,<br>10D07Z6*, 0W8NXZZ*,<br>10D07Z6*, 10D07Z8*,<br>10D07Z7*,
Caesarean delivery	649.8, 669.70, 669.71, 740*,<br>741*, 742*, 744*, 7499*	O82, O75.82<br>10D00Z0*, 10D00Z1*,<br>10D00Z2*
Labouring Caesarean	660.2-660.4, 660.6-660.9, 661,<br>662, 663.0-663.4,663.8, 663.9,<br>669.0, 669.01, 669.9	O60-O64.5, O64.8, O64.9,<br>O65, O66, O68, O69.0, O69.3,<br>O69.5-O69.9, O75-O77
Repeat caesarean	654.2	O34.2
**Exclusion Criteria**		
Ectopic and molar pregnancy and other pregnancies with abortive outcomes	630-639<br>6901*, 6951*, 7491*, 750*	O00, O01, O02, O03, O04,<br>O07, O08, Z33.2<br>10A07ZZ*, 10A08ZZ*,<br>10A00ZZ*, 10A03ZZ*,<br>10A04ZZ*, 10A07ZX*
Multiple Births	V27.2-V27.7, 651	Z37.2-Z37.7, O30
**Cardiovascular disease (any)**	398.91, 402, 410-414, 425,<br>427, 428, 430-438, 440-449	I09.81, I11, I20-I25, I42, I46-I50, I60-I70
**Heart disease (any)**	398.91, 402, 410-414, 425,<br>427, 428, 440-449	I09.81, I11, I20-I25, I42, I46-I50, I70
Ischaemic heart disease	410-414	I20-I25
Atherosclerotic heart disease	440-449	I70
Acute myocardial infarction	410	I21, I22
Hypertensive heart disease	402	I11

Heart failure	428	I50
Cardiomyopathy	425	I42
Cardiac arrythmias	427	I46-I49
**Stroke (any)**	430-438	I60-I69
Ischaemic stroke	430-432	I60-I62, I69
Haemorrhagic stroke	433-437	I63, I65-I67
**Comorbidities**		
Gestational diabetes	648.83	O24, O99.81
Chronic hypertension	642.00-642.24	O10
Gestational hypertension	642.30-642.34	O13
Preeclampsia without severe features	642.40-642.49	O14.0, O14.9
Preeclampsia with severe features	642.50-642.54	O14.1, O14.2
Eclampsia	642.60-642.64	O15
Chronic hypertension with superimposed preeclampsia	642.70-642.74	O16
Placental abruption	641.2	O45
Fetal Growth Restriction/ Small for Gestational Age	656.5, 764.9, 764	O36.5, P05.0, P05.1, P05, P07
Preterm delivery	644.21, 765.2	O60, Z3A.20-Z3A.36
Stillbirth	656.4	O36.4XX

Supplemental Table 2

Time period analysis of risks of cardiovascular disease hospitalisation at 0-29, 30-59, 60-89, 90-179, and 180-365 days following delivery in relation to caesarean delivery (compared to vaginal delivery): Nationwide Readmissions Database, 2010-2018

Cardiovascular disease	Cumulative adjusted hazard ratio (95% confidence interval) of CVD rehospitalisation			
	**30-59 days postpartum**	**60-89 days postpartum**	**90-179 days postpartum**	**180-365 days postpartum**
**Mortality**				
All-cause	1·29 (0·89-1·87)	1·26 (0·84-1·89)	1·19 (0·87-1·62)	1·26 (0·83-1·92)
Cardiovascular disease	1·28 (0·85-1·93)	0·95 (0·57-1·58)	1·22 (0·83-1·81)	0·85 (0·51-1·41)
Heart disease	1·25 (0·79-1·98)	1·00 (0·59-1·71)	1·25 (0·83-1·88)	0·85 (0·49-1·49)
Stroke	1·64 (0·75-3·55)	0·21 (0·05-0·93)	2·52 (1·08-5·89)	0·63 (0·21-1·96)
**Non-fatal complications**				
Cardiovascular disease (any)	1·68 (1·57-1·81)	1·73 (1·56-1·92)	1·60 (1·45-1·76)	1·61 (1·45-1·79)
Heart disease (any)	1·73 (1·60-1·87)	1·79 (1·60-2·00)	1·62 (1·46-1·80)	1·55 (1·38-1·74)
Ischaemic heart disease	1·17 (0·95-1·44)	1·93 (1·47-2·53)	1·55 (1·26-1·90)	1·76 (1·35-2·29)
Atherosclerotic heart disease	1·37 (1·01-1·85)	1·65 (1·07-2·54)	1·62 (1·05-2·49)	1·34 (0·86-2·08)
Acute myocardial infarction	1·20 (0·90-1·60)	2·13 (1·39-3·27)	1·43 (1·02-2·00)	1·63 (1·05-2·53)
Hypertensive heart disease	2·26 (1·66-3·07)	1·55 (1·05-2·29)	1·83 (1·13-2·95)	1·09 (0·63-1·90)
Heart failure	1·85 (1·65-2·08)	1·93 (1·60-2·32)	1·67 (1·38-2·02)	1·62 (1·30-2·04)
Cardiomyopathy	1·42 (1·15-1·74)	2·12 (1·66-2·73)	1·76 (1·37-2·25)	1·35 (1·03-1·78)
Cardiac arrythmias	1·66 (1·48-1·86)	1·58 (1·35-1·85)	1·48 (1·30-1·68)	1·49 (1·28-1·74)
Stroke (any)	1·35 (1·16-1·58)	1·38 (1·11-1·71)	1·48 (1·16-1·90)	1·94 (1·53-2·46)
Ischaemic stroke	1·40 (1·16-1·70)	1·31 (1·02-1·69)	1·50 (1·09-2·06)	1·93 (1·48-2·51)
Haemorrhagic stroke	1·38 (1·07-1·77)	1·19 (0·83-1·72)	1·36 (0·99-1·89)	1·52 (1·01-2·27)

Hazards ratios were based on weighted deliveries adjusted for the confounding effects of maternal age, hospital bed size, hospital type, hospital teaching status income quartile, insurance, and year of delivery through the discrete-time Cox proportional hazards regression model.

Supplemental Table 3
Associations between primary and repeat caesarean delivery (in relation to vaginal delivery) and hospitalisations for cardiovascular disease: Nationwide Readmissions Database, 2010-2018

Cardiovascular disease	Adjusted hazard ratio (95% confidence interval)	
	**Primary caesarean delivery (n = 2,087,124)**	**Repeat caesarean delivery (n = 2,466,368)**
**Mortality**		
All-cause	1·45 (1·17-1·79)	1·17 (0·94-1·47)
Cardiovascular disease	1·33 (1·03-1·73)	1·05 (0·83-1·34)
Coronary heart disease	1·30 (0·97-1·74)	1·01 (0·79-1·30)
Stroke	1·75 (1·09-2·82)	1·14 (0·68-1·89)
**Non-fatal complications**		
Cardiovascular disease (any)	1·93 (1·82-2·05)	1·56 (1·47-1·65)
Heart disease (any)	1·99 (1·87-2·13)	1·60 (1·50-1·70)
Ischaemic heart disease	1·48 (1·28-1·71)	1·54 (1·31-1·81)
Atherosclerotic heart disease	1·61 (1·26-2·07)	1·48 (1·16-1·90)
Acute myocardial infarction	1·24 (1·00-1·52)	1·43 (1·17-1·76)
Hypertensive heart disease	2·44 (1·91-3·10)	1·58 (1·20-2·09)
Heart failure	2·41 (2·19-2·67)	1·61 (1·45-1·80)
Cardiomyopathy	2·02 (1·70-2·39)	1·46 (1·19-1·80)
Cardiac arrythmias	1·65 (1·51-1·79)	1·60 (1·49-1·72)
Stroke (any)	1·48 (1·28-1·72)	1·29 (1·15-1·44)
Ischaemic stroke	1·53 (1·30-1·79)	1·37 (1·20-1·57)
Haemorrhagic stroke	1·20 (0·97-1·47)	1·17 (0·99-1·39)

Hazards ratios were based on weighted deliveries and adjusted for the confounding effects of maternal age, hospital bed size, hospital type, hospital teaching status income quartile, insurance, and year of delivery through the Cox proportional hazards regression model.

Supplemental Table 4
Associations between labouring and non-labouring caesarean delivery (in relation to vaginal delivery) and hospitalisations for cardiovascular disease: Nationwide Readmissions Database, 2010-2018

Cardiovascular disease	Hazard ratio (95% confidence interval)	
	Laboring caesarean delivery (n = 1,850,396)	Non-laboring caesarean delivery (n = 2,701,510)
**Mortality**		
All-cause	1·18 (0·91-1·52)	1·38 (1·14-1·69)
Cardiovascular disease	1·17 (0·85-1·61)	1·20 (0·96-1·49)
Coronary heart disease	1·15 (0·82-1·61)	1·16 (0·91-1·48)
Stroke	1·26 (0·68-2·36)	1·57 (0·99-2·47)
**Non-fatal complications**		
Cardiovascular disease (any)	1·62 (1·53-1·71)	1·82 (1·71-1·93)
Heart disease (any)	1·68 (1·58-1·78)	1·87 (1·76-1·99)
Ischaemic heart disease	1·24 (1·07-1·45)	1·67 (1·45-1·94)
Atherosclerotic heart disease	1·18 (0·87-1·58)	1·74 (1·40-2·17)
Acute myocardial infarction	1·14 (0·93-1·41)	1·45 (1·19-1·77)
Hypertensive heart disease	1·98 (1·52-2·58)	1·93 (1·49-2·50)
Heart failure	1·98 (1·80-2·18)	2·01 (1·81-2·23)
Cardiomyopathy	1·46 (1·23-1·74)	1·92 (1·60-2·31)
Cardiac arrythmias	1·44 (1·33-1·56)	1·75 (1·61-1·89)
Stroke (any)	1·30 (1·51-1·48)	1·44 (1·26-1·64)
Ischaemic stroke	1·29 (1·13-1·48)	1·55 (1·33-1·81)
Haemorrhagic stroke	1·22 (1·02-1·47)	1·16 (0·96-1·40)

Hazards ratios were based on weighted deliveries and adjusted for the confounding effects of maternal age, hospital bed size, hospital type, hospital teaching status income quartile, insurance, and year of delivery through the Cox proportional hazards regression model.

Supplemental Table 5
Bias-corrected associations between mode of delivery and hospitalisations for cardiovascular disease: Nationwide Readmissions Database, 2010-2018

Cardiovascular disease	Bias-corrected rate ratio (95% confidence interval)	
	Hospitalisations up to a year	Hospitalisations 0-29 days
**Mortality**		
All-cause	1·37 (1·28-1·45)	1·48 (1·34-1·62)
Cardiovascular disease	1·18 (1·08-1·30)	1·49 (1·34-1·65)
Heart disease	1·14 (1·04-1·26)	1·39 (1·23-1·57)
Stroke	1·34 (1·17-1·55)	1·76 (1·70, 1·82)
**Non-fatal complications**		
Cardiovascular disease (any)	1·47 (1·40-1·55)	1·59 (1·51-1·66)
Heart disease (any)	1·51 (1·43-1·58)	1·65 (1·57-1·71)
Ischaemic heart disease	1·41 (1·33-1·49)	1·43 (1·34-1·52)
Atherosclerotic heart disease	1·29 (1·21-1·38)	1·49 (1·39-1·60)
Acute myocardial infarction	1·33 (1·24-1·43)	1·24 (1·18-1·31)
Hypertensive heart disease	1·79 (1·72-1·86)	1·77 (1·69-1·85)
Heart failure	1·65 (1·58-1·72)	1·77 (1·70-1·83)
Cardiomyopathy	1·51 (1·43-1·59)	1·70 (1·62-1·77)
Cardiac arrythmias	1·37 (1·30-1·45)	1·53 (1·45-1·61)
Stroke (any)	1·23 (1·16-1·31)	1·19 (1·12-1·26)
Ischaemic stroke	1·27 (1·20-1·35)	1·25 (1·18-1·34)
Haemorrhagic stroke	1·15 (1·08-1·23)	1·06 (0·99-1·14)

Biases due to selection and unmeasured confounding were simultaneously corrected. For details, please refer to the section on statistical analysis in the manuscript.

Figure Legend

Supplemental Figure 1 Kaplan-Meier curve depicting the association of cardiovascular disease-related morbidity with caesarean delivery over time. Time measured in person-months.

Supplemental Figure 2 Kaplan-Meier curve depicting the association of cardiovascular disease-related mortality with caesarean delivery over time. Time measured in person-months.

Supplemental Figure 3 Infographic depicting study population and result. CVD = Cardiovascular disease, HR = hazard ratio.

Supplemental Figure 1

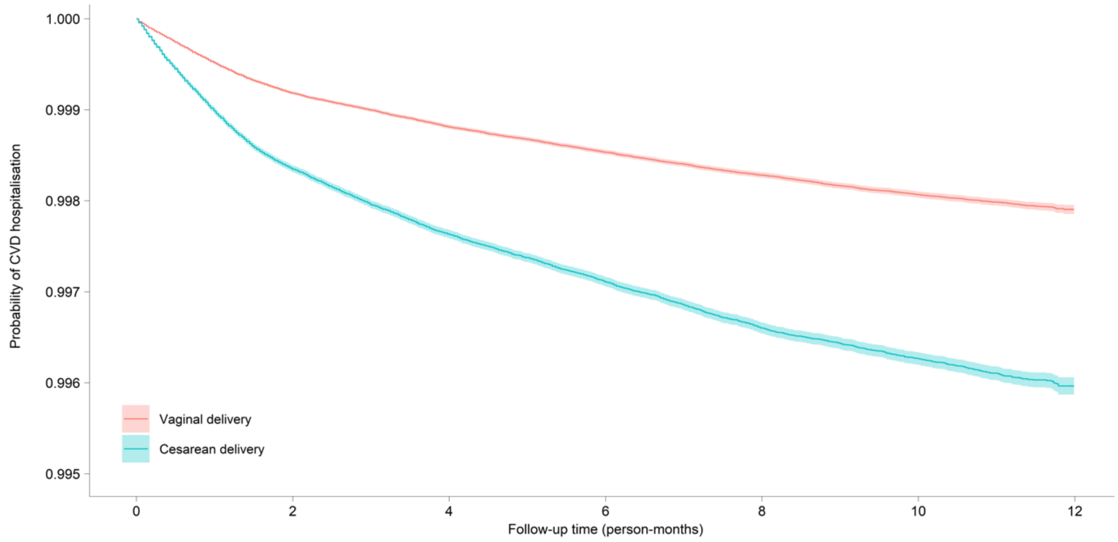

Number at risk (number censored)

	0	2	4	6	8	10	12
Vaginal delivery	9901903 (0)	9019977 (903787)	7158522 (2732428)	5276838 (4612369)	3488718 (6399372)	1735774 (8151741)	0 (9887369)
Cesarean delivery	4769446 (0)	4337259 (438884)	3442371 (1316523)	2540919 (2216407)	1682346 (3073894)	838036 (3917772)	0 (4755674)

Supplemental Figure 2

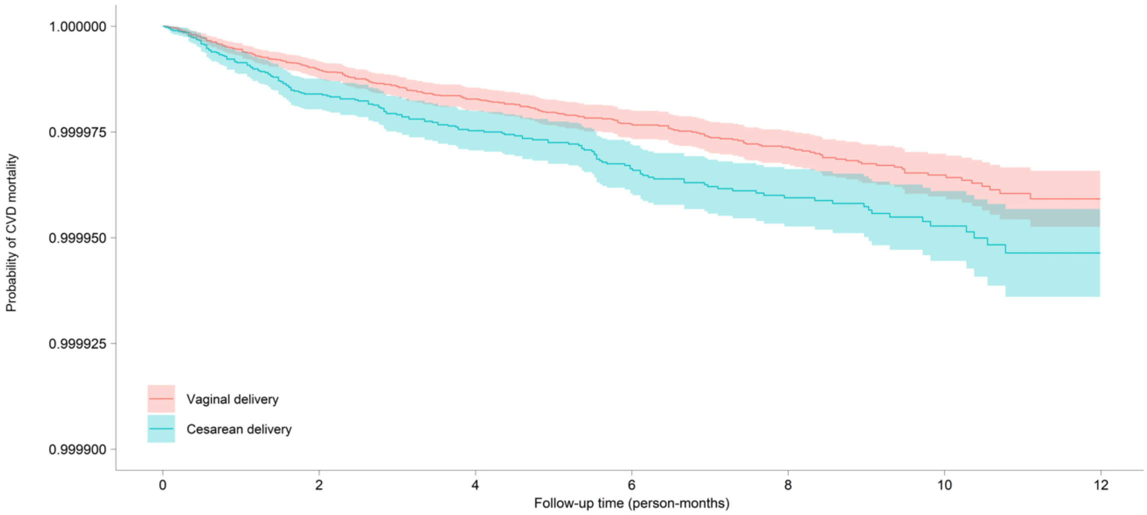

Number at risk (number censored)

Vaginal delivery	9898508 (0)	9029638 (898524)	7170462 (2727889)	5288078 (4610236)	3497599 (6400691)	1740901 (8157371)	0 (9898265)
Cesarean delivery	4765785 (0)	4346779 (433346)	3453908 (1311768)	2551598 (2214052)	1690760 (3074875)	843012 (3922615)	0 (4765623)

Supplemental Figure 3

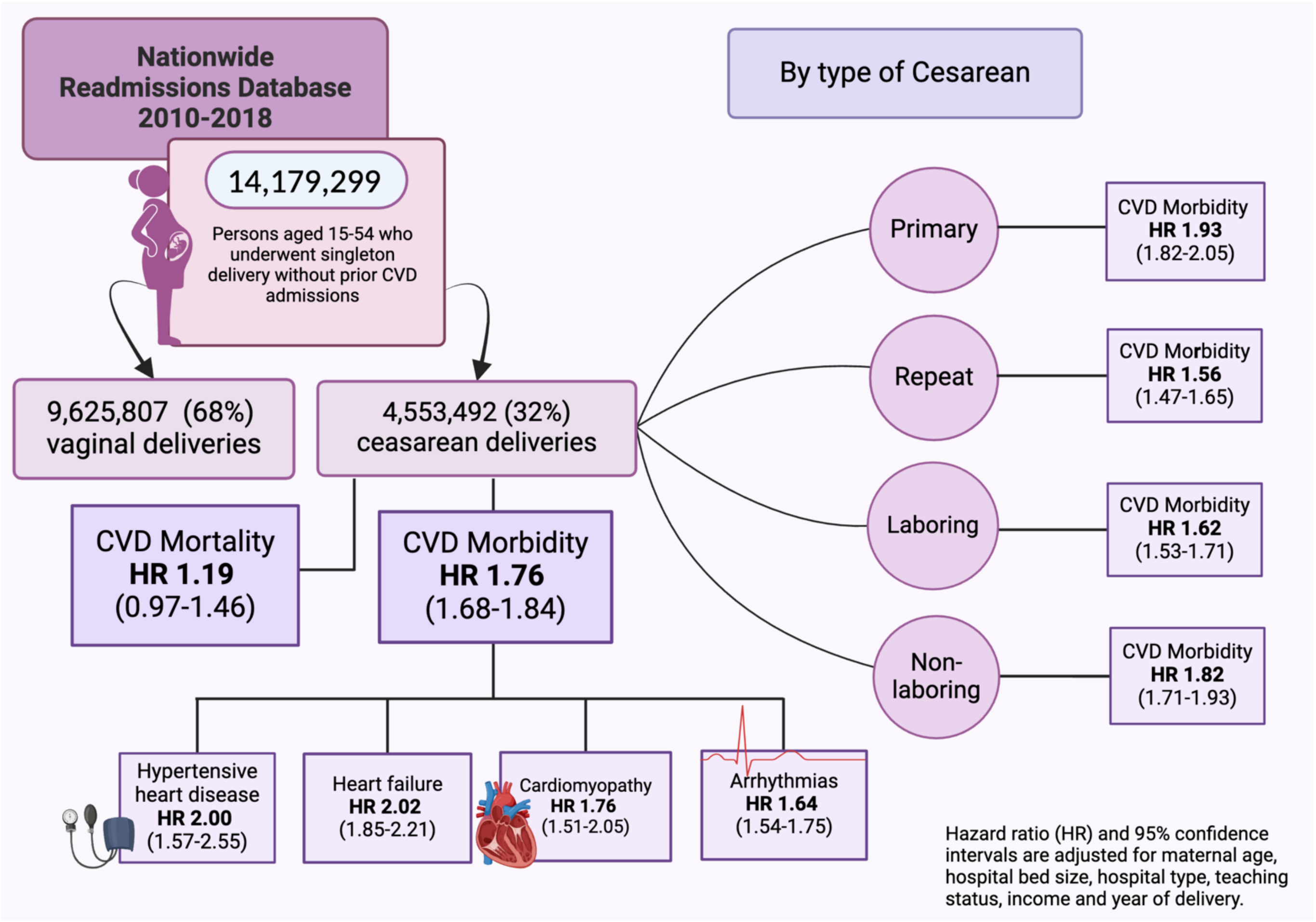

Supplement: Multimedia component 1 [file mmc1.pdf]
